# The Impact of Revascularisation on Quality of Life in Chronic Mesenteric Ischemia

**DOI:** 10.1155/2019/7346013

**Published:** 2019-11-12

**Authors:** J. T. M. Blauw, H. A. M. Pastoors, M. Brusse-Keizer, R. J. Beuk, J. J. Kolkman, R. H. Geelkerken, for the Dutch Mesenteric Ischemia Study Group

**Affiliations:** ^1^Department of Vascular Surgery, Medical Spectrum Twente, Enschede, Netherlands; ^2^Department of Surgery, Leiden University Medical Center, Leiden, Netherlands; ^3^Medical School Twente, Medisch Spectrum Twente, Enschede, Netherlands; ^4^Department of Gastroenterology, Medisch Spectrum Twente, University Medical Center Groningen, Enschede, Netherlands; ^5^Multimodality Medical Imaging M3i Group, Faculty of Science and Technology, Technical Medical Centre, University of Twente, Enschede, Netherlands; ^6^Medisch Spectrum Twente (t.n.v. R.H. Geelkerken, Backoffice Chirurgie B11), Postbus 50.000, 7500 KA, Enschede, Netherlands

## Abstract

**Background:**

Chronic mesenteric ischemia (CMI) is characterized by long-standing abdominal symptoms due to insufficient mesenteric circulation. Data on the effect of revascularisation on quality of life (QoL) for CMI are scarce. This study is the first to evaluate the impact of revascularisation on quality of life.

**Methods:**

Seventy-nine patients with CMI or acute-on-chronic mesenteric ischemia (AoCMI) underwent an intervention of one or more mesenteric arteries between January 2010 and July 2012. QoL before and after intervention was measured with the EuroQol-5D. Preintervention questionnaires were of standard care. Postintervention data were obtained by resending a questionnaire to the patients between February and May 2013. To investigate the clinical relevance of our findings, the minimal clinically important difference (MCID) was used. Since there is no established MCID for CMI, we used the literature reference MCID of inflammatory bowel syndrome (IBS) of 0.074.

**Results:**

Fifty-five (69.6%) of 79 patients returned their questionnaire and 23 (29.1%) were completely filled out. There was a significant increase of the median EQ-index score from 0.70 to 0.81 (*p*=0.02) and a significant reduction of symptoms in the domains usual activities (34.4%) and pain/discomfort (32.3%). There was a significant improvement of 17% in overall current health condition (VAS) (*p*=0.001). The MCID between baseline and postoperative EQ-5D index score was 0.162, indicating a clinically relevant improvement of quality of life after revascularisation.

**Conclusion:**

Quality of life of CMI patients is improved after mesenteric artery revascularisation.

## 1. Introduction

Chronic mesenteric ischemia (CMI) is characterized by chronic abdominal pain resulting from ischemia and caused in most cases by significant stenosis in at least two mesenteric arteries, although an isolated stenosis can be symptomatic as well [1]. Complaints include postprandial pain, weight loss, nausea with or without vomiting, and diarrhoea [1–4]. Typically, patients eat small meals, 6–8 times a day, low in calories and develop a fear of eating, which leads to unintentional weight loss and malnutrition [5].

The etiology of the mesenteric artery stenosis is diverse, including atherosclerosis [1, 6] and external compression by median arcuate ligament syndrome (MALS) [1, 7].

Revascularization is indicated in patients with multivessel stenoses and otherwise unexplained abdominal complaints [1] or in single-vessel stenosis with typical complaints and proven ischemia. Nowadays, the treatment of choice is endovascular antegrade [1, 8–12] or retrograde [13] stenting in case of atherosclerotic intraluminal stenosis and celiac artery release in case of MALS [1, 7].

In chronic pain patients, it has been shown that abdominal symptoms often lead to psychological effects [14], including a high prevalence of depression. Both pain and depression reduce the quality of life and, therefore, reduce health-related quality of life (HRQoL) compared to unaffected individuals [14, 15].

HRQoL instruments are increasingly used to measure patients' health status and to evaluate the effectiveness of health-care interventions [16]. However, the effect of treatment on physical and psychological well-being in CMI patients is still an unexplored frontier [1]. The aim of this study was to measure the impact of revascularisation on HRQoL in patients with CMI.

## 2. Methods

A retrospective analysis was performed to investigate the impact of revascularisation of the mesenteric arteries in patients with CMI on HRQoL. Consecutive patients with CMI and acute-on-chronic mesenteric ischemia (AoCMI) admitted to our tertiary referral centre for treatment of mesenteric ischemia between January 2010 and July 2012 were eligible for inclusion. The clinical symptoms were evaluated by a multidisciplinary group, including a gastroenterologist, interventional radiologist, and vascular surgeon, as previously reported [17]. The inclusion and exclusion criteria are listed in Table 1.

Relevant clinical characteristics, including HRQoL data of patients with CMI measured by the EuroQol-5D (EQ-5D) questionnaire [18], were prospectively registered in our vascular database. All patients were asked to complete this EQ-5D questionnaire during their first hospital admission. Between February and May 2013, all CMI and AoCMI patients who underwent revascularisation between January 2010 and July 2012 were asked to fill in a second EQ-5D questionnaire to collect postinterventional data. The Medical Ethics Committee Twente judged that no further judgement of the study protocol by the committee was required nor was an informed consent procedure necessary, according to the Dutch law on scientific medical research in humans.

### 2.1. Outcome Measures

The EQ-5D is a five-dimensional health state classification consisting the domains mobility, self-care, usual activities, pain/discomfort, and anxiety/depression. Each domain is assessed by a single question on a three-point ordinal scale (no, some, or extreme problems). The EQ-5D also contains self-rating on a 20-centimetre visual analogue scale (VAS), anchored with 100 (“best imaginable health state”) at the top and 0 (“worst imaginable health state”) at the bottom (EQ-VAS) [18].

The EQ-5D index score can be regarded as a continuous outcome scored on a −0.59 to 1.00 scale, with 1.00 indicating “full health” and 0 representing dead. The negative scores represent certain health states valued worse than dead [18]. The EQ-5D index score could be calculated only in completed questionnaires.

Since interpretation in HRQoL scores raises many issues, a minimal clinically important difference (MCID) for the EQ-5D has been developed in order to allow clinicians to make meaningful interpretations of the effect of their treatment. It was defined as “smallest difference in score in the domain of interest which patients perceive as beneficial and which would mandate, in the absence of troublesome side effects and excessive cost, a change in the patient's management” [19]. These are derived from comparison of scores from complex, calculated scores to “simple” clinical outcomes (improved or disappeared pain for example) and result in a minimal difference in score outcome that corresponds with important clinical benefit and, thus, changes via a clinical intervention that is meaningful for the patient [20]. In essence, it links an increase of the EQ-5D with patient relevant outcomes, usually improved or greatly improved on a Likert scale, the so-called anchor of the MCID. There has not been an established MCID for CMI until this day. So, for this study, we used the literature for diseases with corresponding abdominal complaints, such as irritable bowel syndrome (IBS). The MCID for the EQ-5D in one study ranged from 0.011 to 0.140, with a mean of 0.074; we have chosen this as a reference point for the comparisons in our study [21].

### 2.2. Definitions

*CMI* is defined as symptoms of mesenteric ischemia for more than 3 months [1]. The typical presentation includes postprandial pain, weight loss due to fear of eating, or unexplained diarrhoea [22, 23]. AoCMI is defined as acute mesenteric ischemia in patients who previously had typical complaints of CMI [1]. Often, the complaints of CMI worsened over the preceding weeks with prolonged and more severe pain periods, pain even without eating, onset of diarrhoea, or inability to eat at all [22, 23]. *MALS* is defined as epigastric or postprandial pain and weight loss due to external compression of the coeliac artery by the median arcuate ligament [1]. *Technical success* (based on the intention to treat) is defined as successful completion of the procedure and <30% residual stenosis at the end of the procedure [22, 23]. *Primary patency* is defined as uninterrupted patency without the need for any additional procedures [22, 23]. *Clinical success* is defined as uninterrupted relief or improvement of presenting symptoms with a patent revascularized target vessel [22, 23].

### 2.3. Statistical Analyses

Baseline characteristics are displayed as mean with standard deviation or median with differences in pre- and postoperative EQ-5D index score, and scores per domain and EQ-VAS were analysed using the paired *t*-test or Wilcoxon signed-rank test, as appropriate. For categorical variables (domains), these differences were tested with the McNemar test. Differences in baseline characteristics between responders and nonresponders were tested with either an independent *T*-test or Wilcoxon rank sum test for continuous variables and with the chi-squared or Fisher's exact test for categorical variables. Data were analysed using SPSS 21.0.

## 3. Results

**Figure 1:**
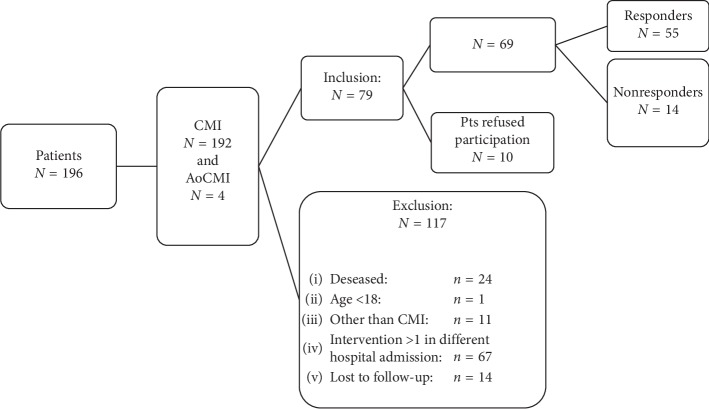
Flowchart of patient inclusion.

Between January 2010 and July 2012, 196 consecutive patients were treated for CMI (192 patients) or AoCMI (4 patients). Seventy-nine patients with CMI or AoCMI met the inclusion criteria for participation (Figure 1).

Fifty-five patients (69.6%) answered the postintervention questionnaire and were included in the data analysis.  Table 2 shows the patient characteristics of these 55 patients. Their mean age was 58 years and 36 (65.5%) were female. Thirty-three patients (60%) underwent endovascular treatment. Fifty-two patients (94.5%) were suffering from CMI. Three patients (5.5%) were diagnosed with AoCMI. Twenty-five patients (45.5%) were diagnosed with single-vessel CMI. Nineteen of these 25 patients (76%) were diagnosed with MALS. Seventeen of them underwent a retroperitoneal endoscopic coeliac artery release and two responders underwent an open transabdominal release. The remaining six of the 25 patients underwent endovascular stenting because of single-vessel intraluminal atherosclerotic stenoses. Thirty patients (54.5%) were diagnosed with multivessel (two or three affected mesenteric arteries) intraluminal stenosis. Twenty-seven of them underwent antegrade and one retrograde endovascular stenting. The remaining two patients underwent antegrade autologous reversed VSM two vessel bypass revascularisation. The mean time between hospital admission and postoperative measurements (*n* = 55) was 20 months (SD ± 8.2).

Technical success was 87%, one-year primary patency was 79%, and one-year clinical success was achieved in 80% of the patients (Table 2).

The 23 patients who completed the questionnaire were significantly younger than the 32 who did not complete the questionnaires (51 ± 19 vs. 63 ± 15 years) (*p*=0.008). Fifteen (65.2%) of those 23 patients had single-vessel CMI compared to 31.2% of those who did not complete the questionnaire (*p*=0.04). The time between the intervention and receiving the questionnaire was significant longer in the completed questionnaire group 22 ± 9 months versus 18 ± 7 months in the incomplete questionnaire group (*p*=0.05). No difference in sex (*p*=0.54) or intervention type (*p*=0.28) between both groups was found. The power to show a difference in EQ-index for the entire group (so *n* = 23) was 0.96 and therefore, above the usual 0.8 that you use for a power analysis.

### 3.1. The EQ-5D Index Score

The EQ-5D index score is shown in Table 3. Twenty-three (41.8%) of 55 patients completed all the 5 domains of the questionnaire after revascularisation. Therefore, there were 32 incomplete questionnaires that were not useful to calculate this index score. Again, preoperative characteristics, treatment, and clinical success of the cohorts' complete and partial responders were matching. The median EQ-5D index score (*n* = 23) increased from 0.70 at baseline to 0.81 (*p*=0.02) after treatment. In three (13%) patients, there was no technical success and their median EQ-5D index score was 0.70 before and 0.67 after intervention (*p*=0.29). The median index score of the twenty patients (87%) in whom technical success was achieved increased from 0.65 to 0.82 (*p*=0.03).

The difference between baseline and postoperative EQ-5D index score was 0.162 (SD ± 0.312, 95%CI 0.027–0.297), which exceeds the range of accepted MCIDs of 0.074 in previous studies [16], indicating a clinically relevant improvement of quality of life after revascularisation.

There was no correlation between neither the EQ-5D index score (*r* = 0.021, *p*=0.88) nor the EQ-VAS score (*r* = −0.091, *p*=0.53) with the time between the intervention and receiving the questionnaire.

### 3.2. Domains EQ-5D

In order to analyse each domain (i.e., pain/discomfort and mobility), only pre- and postoperative measurements of the same patient per domain were useful. Because of missing data, each domain shows a different number of patients.

Table 4 shows the outcomes per domain. Data analysis showed a significant reduction in limitation of daily activities and pain/discomfort (*p* < 0.05) and a numerical reduction of complaints in the domains mobility and anxiety/depression.

### 3.3. EQ-VAS

Table 4 also shows the outcome of the EQ-VAS. Because of missing data, the VAS scores of 31 patients (56.4%) were analysed. The mean VAS-score increased by 17% from 52 at baseline to 68 after intervention (*p*=0.001). The mean VAS score of the 28 patients who underwent an anatomically successful intervention showed a significant increase from 52 to 69 (*p*=0.001), but the mean VAS score of the three remaining patients who did not have an anatomically successful intervention showed a comparable increase from 49 to 57 (*p*=0.08).

## 4. Discussion

The present study evaluates the effect of CMI treatment on physical and psychological well-being and demonstrates improved quality of life. The latter was shown in three different measures. First, treatment of CMI patients had a clinically relevant beneficial outcome to their health-related quality of life. The increase in median EQ-index of 0.162 exceeds MCIDs found in other studies [21]. Second, our results show a significant improvement of almost 17% in overall current health condition (VAS). Third, we found a significant reduction of symptoms in the domains usual activities and pain/discomfort after treatment.

Our literature search showed 2 studies on quality of life after CMI revascularisation. The first, by Skelly et al. [24], describes psychiatric comorbidities in MALS patients undergoing surgery, trying to determine whether these comorbidities are predictive of patient-reported quality of life outcomes. They concluded that patient-reported quality of life significantly improved after surgical therapy for MALS patients but that a pre-existing psychiatric disorder has a poorer outcome in some domains. In contrast to our study, they focused on MALS patients only. Our data gave a broader perspective on the CMI population. The second study is a retrospective analysis by Wagenhäuser et al. [25]. They evaluated the use of the 36-item health survey (SF-36) questionnaire as a tool to investigate HRQoL after revascularisation in CMI patients. They analysed questionnaires of 32 out of their 100 patients, dealing with the same issues we encountered. They showed that CMI patients consider their physical and mental health inferior to the normal German population. However, they did not describe how patients experienced their quality of life before revascularisation. Therefore, it is not possible to assess whether revascularisation has led to improvement of HRQoL and, thus, if revascularisation has a positive effect on HRQoL.

We could not identify any studies on the quality of life in CMI patients using the MCID. Consequently, we cannot compare our data with CMI studies and turned to studies on diseases with abdominal complaints, such as irritable bowel syndrome (IBS). Gralnek et al. [26] showed HRQoL (using the short-form 36 questionnaire) in IBS patients is lower than that of the U.S. general population, for example, patients with gastroesophageal reflux disease or diabetes. Furthermore, it has been shown that HRQoL in IBS patients, using an IBS-specific QoL questionnaire, significantly improved after medicinal treatment [27, 28]. Wang et al. [29] showed that IBS was significantly associated with four of the five EQ-5D dimensions (except self-care; *p*=0.77), which is in line with our findings.

The EQ-5D was developed as an alternative for the very large, and time-consuming, SF-36. The EQ-5D as a measure of HRQoL has been reported for ulcerative colitis (UC) and Crohn's disease (CD). Gibson et al. [30] showed that the mean EQ-5D for 175 UC patients' scores was greater for patients in remission (0.81) than for patients with active disease (0.72). In a large German study by Stark et al. [31], the EQ-5D was said to be “valid, reliable, and responsive in the Inflammatory Bowel Disease (IBD) population studied.” They showed that EQ-VAS and EQ-index scores improved after treatment and that there was a significant difference between the index for active disease and remission. Probert et al. [32] used the EQ-5D in patients with CU before and after medicinal treatment (and with or without placebo). They showed a significant improvement in the domains mobility, usual activity, and anxiety/depression [32]. Another study on patients with CU shows that 90% of the responders report their general health situation to be better after surgery than before [33]. And patients with CU scored better in the domains pain/discomfort and anxiety/depression after surgical treatment than the group of patients receiving medicinal treatment [34]. The mean VAS in these surgically intervened patients was 80.9, which is higher than our results (mean VAS postoperative 68.2, *n* = 31).

The increase in the EQ-5D index score of 0.162 is in line with similar published data on EQ-5D changes after treatment. In a report on 11 studies on the MCID of EQ-5D in various diseases, ranging from IBS to leg ulcers, a mean MCID of 0.074 was reported [21]. It may be difficult to translate outcome of leg ulcers to abdominal pain in CMI. Still, in one of these 11 studies, 161 IBS patients were studied, with an established MCID of 0.065 [21]. Luo et al. [35] showed that the MCID for the EQ-5D (United Kingdom) ranged from 0.036 to 0.204, with the mean being 0.082. Our MCID range of 0.050–0.084 for EQ-5D was within the range of MCID estimates of other disease states. In general, patients who have severe disability had higher MCIDs than patients who had mild-moderate disability. Additional analysis to verify these EQ-5D health status index MCID estimates in an independent dataset should be performed.

The small number of patients (*n* = 3) with an anatomical unsuccessful revascularisation or success not confirmed by diagnostic imaging scored a numerical reduction of complaints in the domains “usual activities” and “anxiety/depression” and a significant increase in VAS of 7.4%, beyond expectation. Also, our results show an increase in median EQ-5D index score, even in patients who had postintervention complications or recurrence of complaints, although we found no statistical significance. However, an explanation of this increase could be that postintervention complications are no longer present and the complaints are less than before intervention.

There are a couple of limitations to our study. First, a potential bias could be the time between treatment and completion of the questionnaire, with more pronounced effects shortly after the treatment. The mean time between revascularisation and completing the postoperative questionnaire (*n* = 55) was 20 months (SD ± 8.2). We found no correlation between neither the EQ-5D index score (*r* = 0.021, *p*=0.88) nor the EQ-VAS score (*r* = −0.091, *p*=0.53) with the interval time. Second, only 79 of 196 patients (40.3%) were eligible for inclusion. Third, of 55 patients responded, in 32 questionnaires, one or more questions were missing. It is unclear why questions were not answered. It did not concern a specific domain. When we analysed the missing questions, they were evenly distributed over the domains and between potential outcomes (responders, partial responders, and nonresponders). We therefore think these missing data do not represent an important bias on the outcomes. Fourth, we cannot rule out a placebo effect. Ideally, an RCT including a sham intervention cohort should be performed to see QoL-related improvement after therapy. However, given the severity of symptoms and available literature data, it is unlikely that such an RCT study will ever be performed in patients with severe chronic mesenteric ischemia.

In conclusion, this study is the first to demonstrate improvement on quality of life in CMI patients after mesenteric artery revascularisation, measured with the EQ-5D. The measured differences are in line with other studies on treatment of abdominal complaints. Prospective research should follow this retrospective study to limit the chance of missing data. It can help us to better identify these patient groups by including larger subgroups of patients in order to study differences between subgroups. Establishing the MCID as a disease-related quality of life measurement instrument for mesenteric ischemia in order to provide more detailed information about this category of patients both for better understanding the patient's expectations as well as future studies on treatment effects can also be investigated.

## Figures and Tables

**Table 1:** Inclusion and exclusion criteria.

Inclusion criteria
Patients aged 18 + years
Mastering the Dutch language, living in the Netherlands
Chronic mesenteric ischemia or acute-on-chronic mesenteric ischemia
Underwent one or more interventions for CMI in one hospital admission between 01-01-2010 and 01-07-2012
Exclusion criteria
Deceased between hospitalization and second EQ-5D measurement
Acute mesenteric ischemia (AMI)
Lost-to-follow-up
Underwent multiple interventions of one of more mesenteric arteries related to CMI in different hospital admissions
Only diagnostic procedure, no revascularisation performed

**Table 2:** Patient characteristics of responding patients.

	Patients, *n* = 55
Age at hospital admission (years), mean ± SD	58 ± 17.5
Time between hospital admission and postoperative measurement (months), mean ± SD	19.6 ± 8.2
Gender, *n* (%)	
Man	19 (34.5)
Woman	36 (65.5)
Complication/recurrent complaints, *n* (%)	
During hospital admission	9 (16.4)
After hospital admission	15 (27.3)
Intervention type, *n* (%)	
Endovascular	33 (60.0)
Open repair	5 (9.1)
AC release	17 (30.9)
x-vessel disease, *n* (%)	
1	25 (45.5)
2	14 (25.5)
3	16 (29.0)
CMI vs. Acute-on-chronic, *n* (%)	
Chronic	52 (94.5)
Acute-on-chronic	3 (5.5)
Technical success, *n* (%)	48 (87.3)
Primary patency	30 out of 38 (79)
Clinical success	44 out of 55 (80)

**Table 3:** EQ-5D index score before and after revascularisation.

	Before	After	*p*
Total (*n* = 23)	0.70 (0.43–0.77)^1^	0.81 (0.67–0.93)	*p*=0.02
AS^2^ (*n* = 20)	0.65 (0.38–0.77)	0.82 (0.77–0.93)	*p*=0.03
AU^3^ (*n* = 3)	0.70 (0.60–0.70)	0.67 (0.67–0.81)	*p*=0.29

^1^Given median IQR. ^2^Anatomical success, confirmed by diagnostic imaging. ^3^Anatomical unsuccessful and/or not confirmed by diagnostic imaging.

**Table 4:** EQ-5D scores per domain and EQ-VAS score.

Domain	All, *n* = 55	
	Pre	Post
Number (%)		
Mobility, *n*^1^		
No problems	16 (50.0%)	19 (59.5%)
Some problems	15 (46.8%)	13 (40.5%)
Extreme problems	1 (3.2%)	—

Self-care, *n*^1^		
No problems	29 (90.6%)	28 (87.5%)
Some problems	3 (9.4%)	4 (12.5%)
Extreme problems	—	—

Usual activities, *n*^1^		
No problems	5 (15.6%)	16 (50.0%)^*∗*^
Some problems	19 (59.4%)	15 (46.9%)
Extreme problems	8 (25.0%)	1 (3.1%)

Pain/discomfort, *n*^2^		
No problems	1 (3.2%)	11 (35.5%)^*∗*^
Some problems	22 (71.0%)	17 (54.8%)
Extreme problems	8 (25.8%)	3 (9.7%)

Anxiety/depression, *n*^3^		
No problems	7 (29.2%)	17 (70.8%)
Some problems	15 (62.5%)	6 (25.0%)
Extreme problems	2 (8.3%)	1 (4.2%)

EQ-VAS “current health condition,” mean (SD)^2^	51.6 (19.7)	68.2 (17.2)^*∗*^

^1^Missing data in 23 of 55 responders (all: *n* = 32, AS: *n* = 29, NAS: *n* = 3). ^2^Missing data in 24 of 55 responders (all: *n* = 31, AS: *n* = 28, NAS: *n* = 3). ^3^Missing data in 31 of 55 responders (all: *n* = 24, AS: *n* = 21, NAS: *n* = 3) ^*∗*^*p* < 0.05.

## Data Availability

All data are stored according to regulations in the Netherlands. The data are available from the corresponding author upon request.
